# Industry Perceptions and Experiences with the Access Consortium New Active Substance Work-Sharing Initiative (NASWSI): Survey Results and Recommendations

**DOI:** 10.1007/s43441-024-00624-7

**Published:** 2024-03-08

**Authors:** Gaia Geraci, Robert Smith, Alison Hansford, Eric Johnsson, Helen Critchley, Lama Abi Khaled, Laura King, Michelle Cheng, Tanja Colin, Tse Siang Kang

**Affiliations:** 1 Clarivate, 70 St. Mary Avenue, London, EC3A 8BE UK; 2 Association of the British Pharmaceutical Industry (ABPI), Hay’s Galleria, 2nd Floor Goldings House, 2 Hay’s Lane, London, SE1 2HB UK; 3 Accumulus Synergy, 1534 Plaza Lane, Suite #210, Burlingame, CA 94010 USA; 4 Medicines Australia, 17 Denison Street, Deakin, ACT 2600 Australia; 5 Innovative Medicines Canada, 1220-55 Metcalfe St., Ottawa, ON K1P6L5 Canada; 6 Singapore Association of Pharmaceutical Industries (SAPI), 151 Chin Swee Rd., #02-13A/14, Manhattan House, 169876 Singapore; 7 Interpharma, Petersgraben 35, 4051 Basel, Switzerland

**Keywords:** Access consortium, NASWSI, Regulatory, Work-sharing, Registration, Collaboration

## Abstract

The Access Consortium New Active Substance Work-Sharing Initiative, or “Access” for simplicity, allows regulatory authorities (RAs) of the Access Consortium countries to jointly review applications for the registration of new active substances or for new indications. Using a survey developed by the pharmaceutical industry trade associations of the five Access Consortium countries—Australia, Canada, Singapore, Switzerland, and the United Kingdom (UK)—this study gathered insights into the perceptions and experiences of the Access pathway held by affiliates of pharmaceutical companies. Understanding industry perceptions of Access is important for the success of the initiative, as participation is voluntary. Findings indicate that affiliates who participated in Access had mostly positive experiences with this pathway; most affiliates were satisfied with their interactions with the Access RAs and appeared willing to continue to participate in the initiative. Affiliates’ reasons for not having yet participated in Access included a lack of opportunity to do so and perceived barriers, such as the Access pathway being too complicated to manage. Recommendations to improve Access cover six key areas: ensure predictability, increase guidance and transparency, streamline processes, maintain flexibility, increase harmonization, and advance RA-industry cooperation. This study should facilitate informed discussions among relevant stakeholders on how to improve Access to maximize efficiencies, accelerate approvals, and improve patient access to innovative medicines.

## Introduction

The Access Consortium, formerly known as the “ACSS Consortium,” is a coalition of medium-sized Regulatory Authorities (RAs) formed in 2007 by the Therapeutic Goods Administration (TGA) of Australia, Health Canada of Canada, the Health Sciences Authority (HSA) of Singapore, and Swissmedic of Switzerland. In 2018, the first work-sharing pilot took place. In October 2020, the Medicines and Healthcare products Regulatory Agency (MHRA) of the UK joined the coalition, resulting in the Consortium being renamed from “ACSS” to “Access.” The Access Consortium aims to maximize international co-operation and increase the capacity of each RA to ensure timely access to high quality, safe, and effective therapeutic products through international collaboration and work-sharing [1]. The Access Consortium has working groups on various areas, such as: New Active Substances, Generic Medicines, Biosimilars, Collaboration on International Council for Harmonization (ICH), IT Architecture, and, since 2023, Advanced Therapy Medicinal Products (ATMPs). Members of working groups have regular meetings to exchange information on regulatory issues and challenges. The are currently three authorization procedures: the New Active Substance Work-Sharing Initiative (NASWSI), the Biosimilar Work Sharing Initiative (BSWSI), and the Generic Medicine Work Sharing Initiative (GMWSI) [1].

This study focuses on industry perceptions with the NASWSI, hereafter referred to as “Access” for simplicity. By participating in Access, two or more RAs can jointly review applications for the registration of new active substances (NASs), a new chemical entity (NCE) or new biological entity (NBE), and for new indications. This reduces duplication of work and allows for efficient use of resources, while allowing each RA to retain the sovereignty to make independent approval decisions [2].

### The Access Consortium as Part of Collaborative Efforts Among RAs

The traditional approval pathway for therapeutic products can be lengthy and resource intensive for RAs and companies. To promote efficiencies and facilitate timely approval of safe and effective therapeutic products, some RAs are increasingly collaborating, engaging in work sharing, and using reliance in regulatory decision-making (considering decisions of other trusted RAs). Some RAs are also contributing to increased harmonization and regulatory convergence, adopting global regulatory standards, and participating in international fora such as the International Council for Harmonisation of Technical Requirements for Pharmaceuticals for Human Use (ICH); International Coalition of Medicines Regulatory Authorities (ICMRA), and Pharmaceutical Inspection Co-operation Scheme (PIC/S). In this context, an important collaborative initiative is Project Orbis, of which the Access Consortium RAs are also part. Project Orbis is coordinated by the United States (US) Food and Drug Administration (FDA) and provides a framework for concurrent submission and review of oncology products among international partners to facilitate faster patient access to innovative cancer treatments [3]. The Access Consortium, allowing work-sharing among RAs which maintain independence in their decision making, can be considered as a successful example of collaborative efforts among like-minded RAs.

### The NASWSI Process

As outlined in the NASWSI Operational Procedures [2], applications should be submitted simultaneously to at least two RAs, and ideally to more. A standard or a priority review procedure is possible. The work-sharing procedure starts upon submission of an Expression of Interest (EoI) form, in which applicants indicate the review procedure that they intend to use. A technical (scientific) pre-submission meeting between the applicant and each RA, or more RAs, may be conducted as well as a logistical pre-submission meeting to confirm the logistics and expectations related to the requirements, timelines, and processes. For NAS applications, each Common Technical Document (CTD) Module is typically reviewed by a specific RA, and other RAs conduct a peer review of the assessment reports (ARs) for each module. Each RA reviews their relevant CTD Module 1. For new indication applications, usually one RA evaluates Module 5, with the other RAs conducting a peer review of the ARs and evaluating their respective Module 1. The RAs prepare an AR and a List of Questions (LoQ) for the module(s) they are responsible for. The RAs also conduct a peer review of the ARs and LoQ. Following this, each RA sends a consolidated LoQ and a copy of the ARs they have prepared to their local applicant [2]. In the standard procedure, consolidated LoQs are normally used (which consist of questions common to all RAs and country-specific questions), however the RAs may indicate a preference to issue rolling questions for the module they are responsible for. In the priority procedure, rolling questions are usually used. Applicants send the same set of responses to the consolidated LoQ to each RA (responses to country-specific questions for Modules 3–5 are only submitted to the applicable RA, unless differently requested). If there are no outstanding issues following the RAs’ assessment of the responses to the LoQ and a peer review of the AR of the responses, then the report(s) are finalized and the RAs proceed to the national steps, during which each RA makes a final decision. This phase includes the finalization of product labelling (labeling negotiation is conducted nationally) which is likely to differ from one jurisdiction to another, and it may also include expert advisory committee meetings [2].

### Progress so Far

As of June 2023, the NASWSI had approved 25 NAS work-sharing applications, including the first two five-way (including all five RAs) work-sharing applications in 2022 [4]. In addition to reducing duplication of work and facilitating efficient use of resources, Access reviews seem to have a positive impact on approval times. For all the Access RAs (excluding MHRA due to lack of data), the median approval time was faster for NASs approved via Access compared to all NASs approved between 2018 and 2022 (with approval time being calculated from the date of submission to the date of approval by the RAs) [5]. Both NCEs and NBEs have been approved following an Access review, including anti-cancer and immunomodulators, and cardiovascular therapies [6].

### Aims of this Study

This study provides insight into the perceptions and experiences of the Access pathway held by affiliates of pharmaceutical companies who had or had not yet taken part in Access, based on an online survey conducted in 2022 in the five Access Consortium countries. The survey explored potential barriers to participation in Access, the changes required to increase participation, and experiences with the different phases of Access. Other areas of exploration included the impact of participating in Access, the perceived benefits of Access, the impact of the participation in an Access review of specific countries, and affiliates’ views on proposed changes to the pathway. Understanding the industry perceptions of Access is important for the success of the initiative, as participation is voluntary. Industry recommendations to further improve the pathway are provided.

## Methods

The survey was developed by the following pharmaceutical industry trade associations of the five Access Consortium countries: Medicines Australia, Innovative Medicines Canada, the Singapore Association of Pharmaceutical Industries (SAPI), Interpharma (Switzerland), and the Association of the British Pharmaceutical Industry (ABPI). The survey aimed to understand the perceptions and experiences of affiliates of pharmaceutical companies with Access in their respective countries, in relation to innovative therapeutic products (generics were outside the survey scope). The survey was open from 20 September 2022 to 3 November 2022. It was sent by the five trade associations to 164 affiliates via trade association membership email distribution lists. Respondents were asked to complete the survey from the perspective of the local affiliate in the respective country. Only one response per company per local affiliate could be submitted. The online survey included multiple choice and open-ended questions, so both quantitative and qualitative insights were gathered. Anonymized survey results were extracted and shared with Clarivate for the analysis and extrapolation of key insights and recommendations.

## Results

### Survey Respondents

**Fig. 1:**
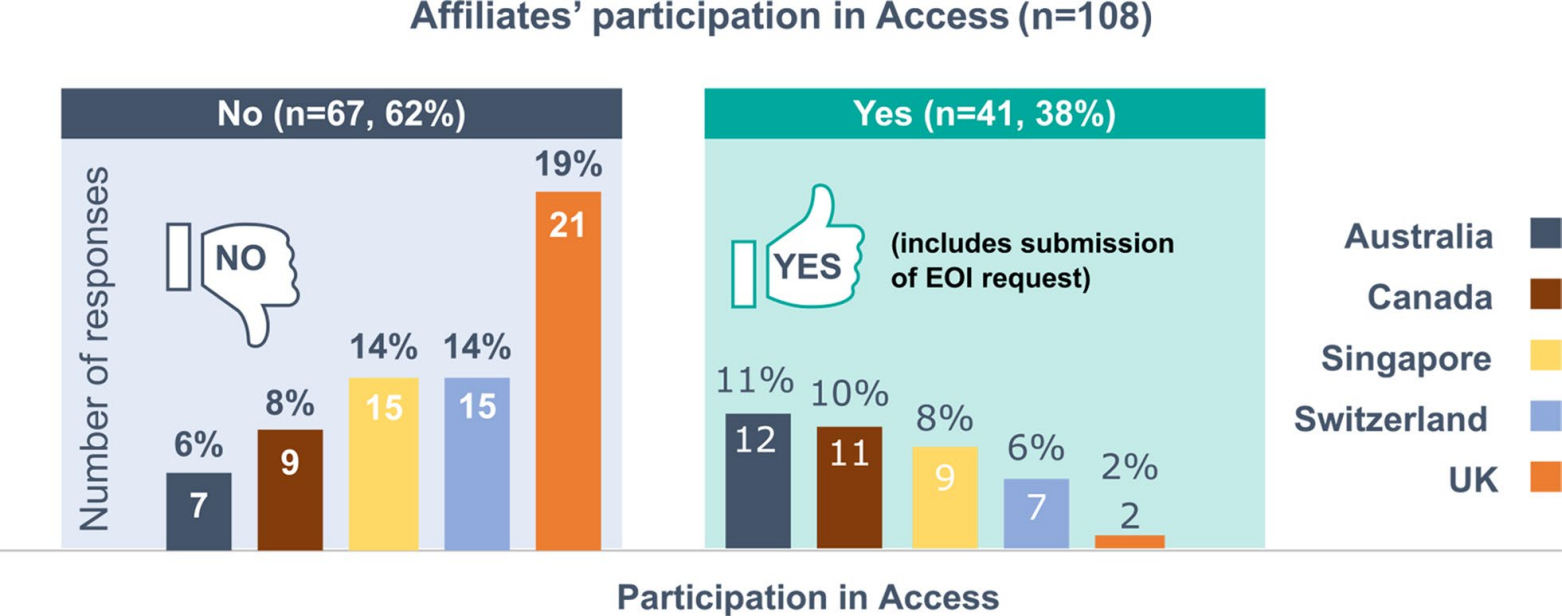
Affiliates’ participation in Access (*n* = 108)

Of the 164 affiliates who were sent the survey, 108 survey responses were received [Australia (*n* = 19); Canada (*n* = 20); Singapore (*n* = 24); Switzerland (*n* = 22); UK (*n* = 23)], resulting in a response rate of 66% (108/164). Figure 1 shows affiliates’ participation in Access (*n* = 108). Two key types of respondents were identified: a majority of affiliates who had not yet taken part in Access (62%, *n* = 67), and a minority of affiliates who had taken part in Access (38%, *n* = 41). Australia had the highest number of affiliates that had participated in Access (*n* = 12). The UK, the latest country to join the Access Consortium, had the lowest number (*n* = 2) (Fig. 1).

### Reasons for not Participating in Access and Changes Suggested by Affiliates

Affiliates who had not yet taken part in Access (*n* = 67, 62%) selected the following reasons for their lack of participation (each affiliate could select more than one option): risk of divergent decisions between participating RAs (39%, *n* = 26); process too complicated to manage (37%, *n* = 25); differences in requirements and review practices between countries (36%, *n* = 24); resources and cost (30%, *n* = 20); and slower approval times compared to national review (22%, *n* = 15). Around half of the respondents (48%, *n* = 32) added “other” reasons for not participating (not listed as response options), including: not having identified a suitable medicinal product for the Access pathway, lack of clear written process guidelines, lack of experience with the process, and absence of the European Medicines Agency (EMA) and US FDA in the initiative. Additionally, two UK affiliates mentioned preference for the “reliance” route—presumably referring to the European Commission Decision Reliance Procedure (ECDRP) introduced after Brexit, which allows the MHRA to rely on a European Commission decision to grant a marketing authorization for a therapeutic product [7].

According to affiliates who had not yet taken part in Access (*n* = 67, 62%), the following changes would be required to encourage participation in Access (several options could be selected): shorter review timelines versus national review (78%, *n* = 52); better alignment of process for priority applications in all countries (75%, *n* = 50); improved guidance on Access pathway and procedures (73%, *n* = 49); joint scientific advice meetings (43%, *n* = 29); and acceptance of conditional/provisional applications (36%, *n* = 24). Other suggested changes (not listed as response options) included: removal of country-specific requirements, provision of training and examples for affiliates, financial incentives such as reduced fees, having a common electronic platform for information sharing, and convergence of approval timelines among RAs so that they are more similar.

### Affiliates’ Experiences with Access

Affiliates who had taken part in Access (*n* = 41, 38%) were asked to share insights on their experience with the different phases of this pathway.

#### The Expression of Interest (EoI) Phase

Overall, most affiliates reported a positive experience with the Expression of Interest (EoI) phase. In particular, 10% (*n* = 4) of affiliates rated their experience with this phase as “excellent,” 66% (*n* = 27) as “good,” 19% (*n* = 8) as “average,” and 5% (*n* = 2) as “bad.” Comments on this phase varied; some affiliates appreciated open dialogue with RAs, swift responses from RAs and the simplicity of the EoI form; while others mentioned feedback from RAs taking longer than expected. Most affiliates (71%, *n* = 29) reported receiving timely feedback from their RA after submitting the EoI form or requesting feedback or advice. The majority of affiliates requested a logistics meeting prior to filing each submission (61%, *n* = 25), with some affiliates commenting that such meetings were useful for the first submission/s, but no longer necessary for subsequent submissions as queries could be addressed via email. Most affiliates (76%, *n* = 31) thought that the evaluation plan, milestones, and review responsibilities among the participating RAs had been made clear to them prior to filing their submission.

#### The Work-Sharing Phase

Affiliates’ experience with the work-sharing phase was also mostly positive; 5% (*n* = 2) of affiliates rated this phase as “excellent,” 73% (*n* = 30) as “good,” 20% (*n* = 8) as “average,” and 2% (*n* = 1) as “bad,” Some affiliates highlighted positive progress in comparison with previous experiences with this phase, progress to a single LoQ, clear timelines, and improvement in guidelines and communication with affiliates. However, some other affiliates reported challenges related to this phase, such as receipt of rolling questions from Health Canada which slowed applications and made timelines unpredictable, non-consolidated questions, and separate country-specific questions.

According to most affiliates (66%, *n* = 27), the process and milestone dates were adhered to during this phase, while a minority (34%, *n* = 14) thought this was not the case, with reasons for deviation from milestones including rolling questions and unexpected rounds of questions.

#### The National Decision-Making Phase

The experience with the national decision-making phase was rated as “excellent” by 5% (*n* = 2), “good” by 54% (*n* = 22), “average” by 29% (*n* = 12), and “bad” by 12% (*n* = 5) of affiliates. Several affiliates stressed their appreciation for the flexibility, responsiveness, and organization of RAs. One affiliate, referring to a specific submission, questioned if the advisory committee meeting was strictly necessary and noted this contributed to a delayed national approval compared to approvals of other RAs.

### The Impact of Participating in Access

Among the affiliates who participated in Access (*n* = 41, 38%), 80% (*n* = 33) thought that approval via Access had had “no impact” on pricing and reimbursement timelines in their country and 5% (*n* = 2) thought that there was “negative" impact. The remaining 15% (*n* = 6) reported a “positive impact,” with some affiliates noting that the HTA (Health Technology Assessment) processes were able to start earlier due to the earlier regulatory approval via Access.

**Fig. 2:**
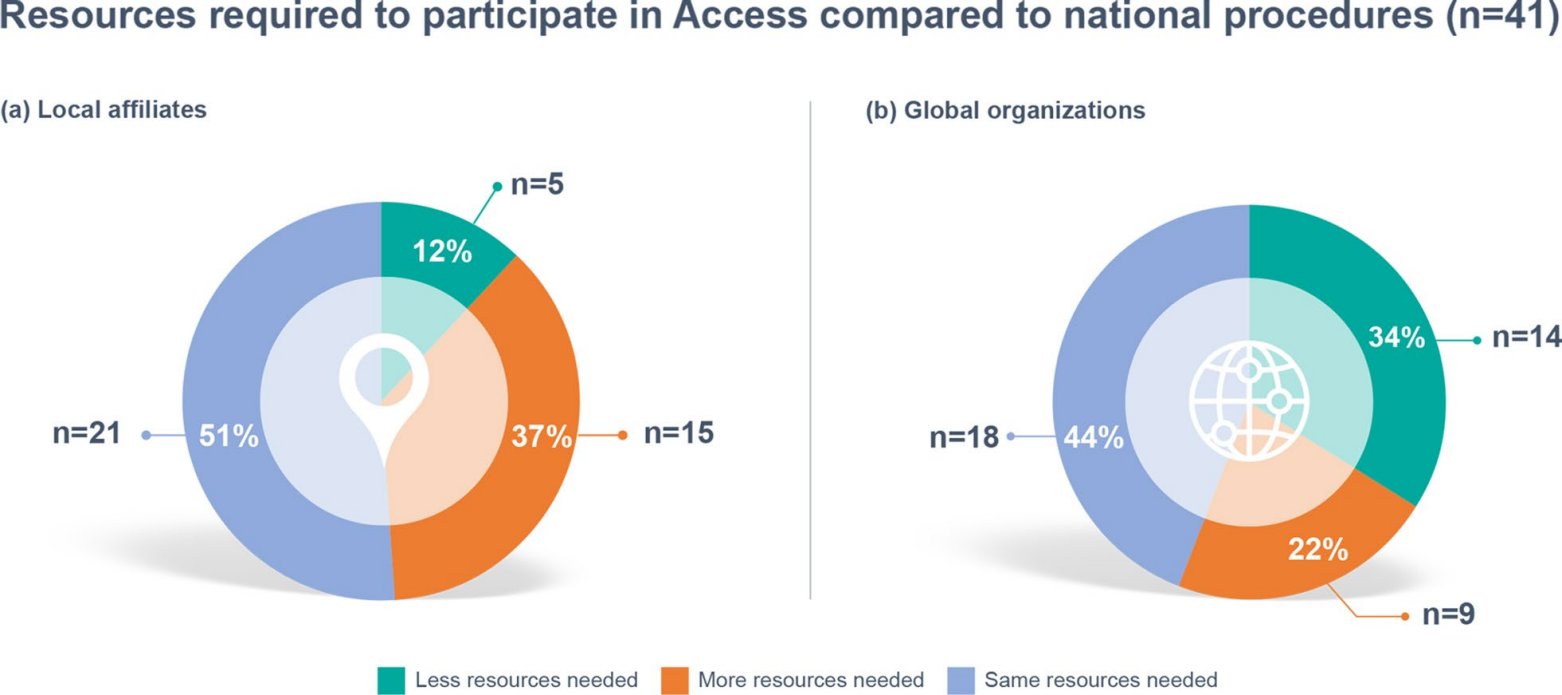
Resources required to participate in Access from **a** local affiliates and **b** global organizations compared to national procedures (*n* = 41)

Affiliates who had participated in Access (*n* = 41, 38%) were asked to outline what impact a review via the Access pathway had had on resource requirements, from the perspectives of both their local affiliates and their global organizations. Figure 2 outlines the resources required from (a) local affiliates and (b) global organizations compared to national procedures (Fig. 2).

Most affiliates believed that the same resources were needed from their local organization (51%, *n* = 21) and their global organization (44%, *n* = 18) when participating in an Access review. Fourteen affiliates (34%) thought that an Access review required “less resources” from their global organization compared to a national procedure, while only 5 affiliates (12%) thought the same was true for their local organization. One affiliate noted that by participating in Access, more resources were required due to additional alignment and communication with other affiliates, while their global organization benefitted more from the reduced number of questions which had been consolidated.

### Perceived Benefits of Access and the Influence of the Participation of Other Countries

**Fig. 3:**
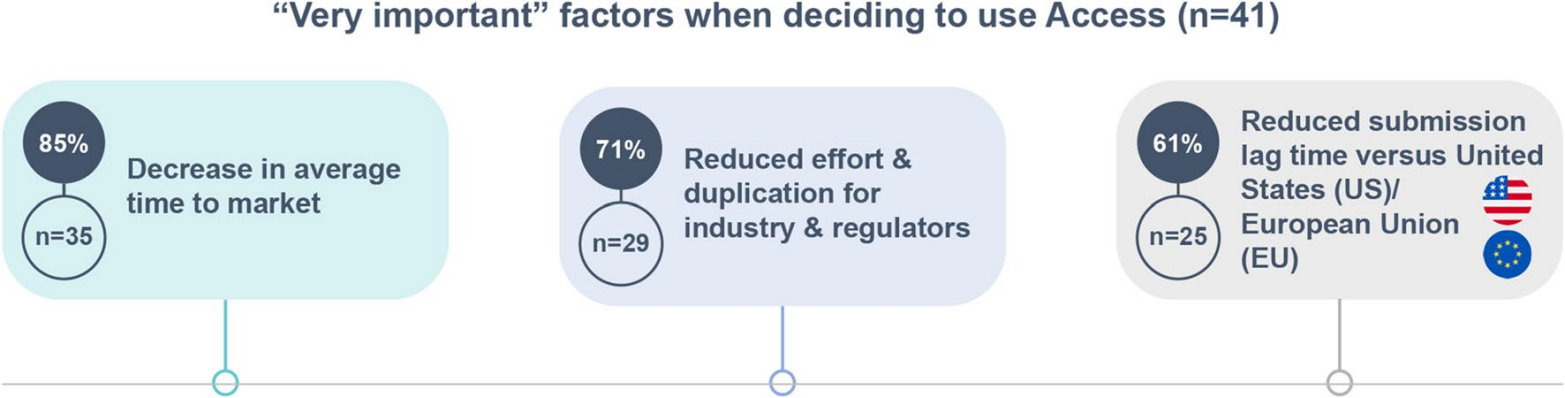
“Very important” factors when deciding to use Access (*n* = 41)

According to affiliates who had participated in Access (*n* = 41, 38%) the pathway offered the following benefits (several options could be selected): gaining experience with an evolving work-sharing pathway (76%, *n* = 31); near simultaneous approval in multiple countries (73%, *n* = 30); shorter review compared to national timeline (61%, *n* = 25); and reduced number of RA questions overall (61%, *n* = 25). Some respondents (34%, *n* = 14) added "other" benefits (not listed as response options), including: ensuring their country remains a tier one country for global submissions, reduced workload for regulatory affairs teams, and receiving RAs questions simultaneously. Affiliates who had participated in Access (*n* = 41, 38%) also rated the importance of three proposed factors when deciding to use the Access pathway: decrease in average time to market; reduced effort and duplication for industry and regulators; and reduced submission lag time versus the US or European Union (EU). Figure 3 outlines the number of affiliates who rated these factors as “very important” (five ratings were possible, ranging from "very important" to "not at all important"), with “decrease in time to market” considered “very important” by the largest proportion of affiliates (85%, *n* = 35) (Fig. 3).

When all affiliates (*n* = 108) were asked if participation of any specific country/countries would *encourage* them to participate in Access, 47% (*n* = 51) of affiliates responded “No” and 53% (*n* = 57) mentioned one or more countries. Among those affiliates who indicated one or more countries (*n* = 57), the majority indicated the UK (91%, *n* = 52), followed by Australia (81%, *n* = 46), Canada (74%, *n* = 42), Switzerland (49%, *n* = 28), and Singapore (35%, *n* = 20). Reasons for mentioning the UK included the MHRA being perceived as being a “lead authority,” the possibility for smaller RAs to learn from them, and its attractive advertised review timeline of 150 days. When asked if participation of any specific country would *discourage* affiliates from participating in Access, the vast majority (*n* = 94, 87%) responded “No.” Among the very few respondents who did mention one or more countries (*n* = 14), some (*n* = 10) indicated Switzerland, followed by Singapore (*n* = 4), the UK (*n* = 3), Canada (*n* = 3) and Australia (*n* = 1).

### Proposed Changes and Recommendations to improve Access

All affiliates (*n* = 108) shared their views on possible changes to Access. Most affiliates (65%, *n* = 70) agreed that there would be a benefit in developing a framework more similar to Project Orbis to allow for sharing of reviews without formal work-sharing. Some of these affiliates commented that Project Orbis allows the RAs to leverage regulatory assessments and to reduce questions to sponsors, potentially contributing to shorter approval timelines (also because the US FDA leads the initiative) and that this framework is helpful for those RAs with resource constraints. Among the affiliates who did not see a benefit in developing a framework more similar to Project Orbis (35%, *n* = 38) some commented that Orbis’ timelines are more unpredictable compared to Access’ timelines and highlighted their preference for the work-sharing typical of Access (as opposed to simple sharing of reviews of Orbis) which leads to reduced workload for RAs and encourages regulatory decisions in the same timeframe.

**Fig. 4:**
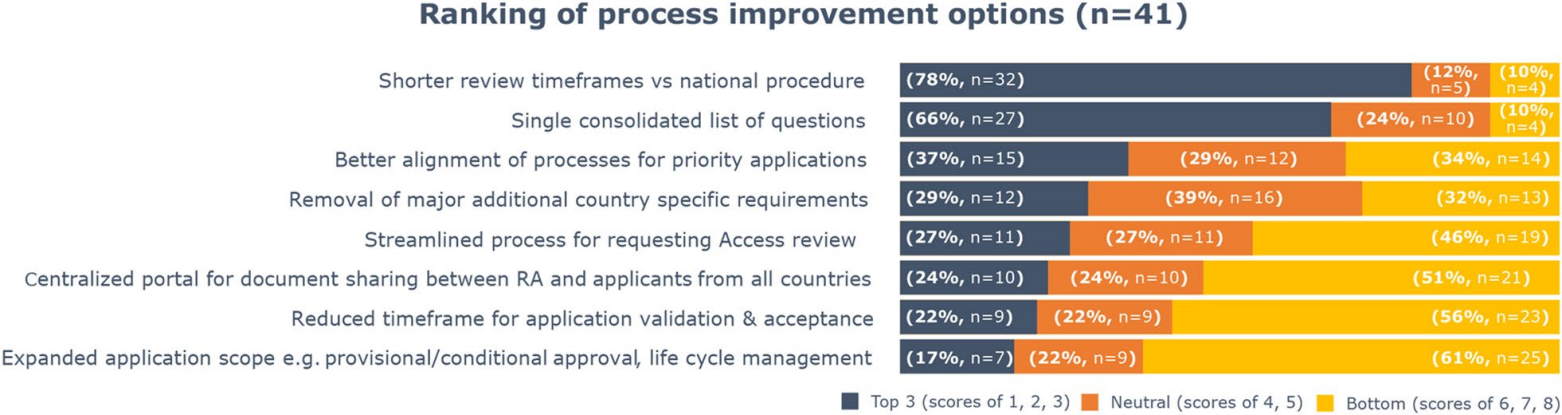
Affiliates’ ranking of Access process improvement options (*n* = 41)

Affiliates who had participated in Access (*n* = 41, 38%) were asked to rank eight proposed process improvement options for the Access pathway, giving scores from 1 to 8 (from the most to the least important). Figure 4 shows the affiliates’ ranking of these options (Fig. 4). ‘Shorter review timeframes vs national procedure’ was ranked by 78% (*n* = 32) of affiliates among their top 3 options, followed by ‘single consolidated list of questions’ (66%, *n* = 27), and ‘better alignment of processes for priority applications’ (37%, *n* = 15). Notably, ‘shorter review timeframes vs national procedure’ was also rated as the single most important improvement by most affiliates (58%, *n* = 24) (not shown on Fig. 4).

According to 94% (*n* = 102) of all affiliates, an Access industry/RA Forum should be established to ensure processes remain fit for purpose in the face of rapidly evolving new technologies. According to affiliates, potential benefits of such a Forum included: allowing the RAs and the industry to engage, sharing of best practices and challenges, increasing transparency, allowing the industry to help to drive actions to improve the initiative, and maintaining the pathway’s agility with treatment advances (such as cell and gene therapy).

Finally, when all affiliates (*n* = 108) were asked if they saw advantages in broadening the scope of Access to full life-cycle management, 63% (*n* = 68) of affiliates agreed. Some affiliates noted that broadening the scope would avoid companies having to submit the same package in multiple countries for the same variation, and that this may increase efficiency and decrease duplication of work. It was also suggested that this could be reserved for complex variations only and that variation requirements should be aligned as much as possible among countries. Reasons for not seeing advantages in broadening the scope of Access to full life-cycle management (37%, *n* = 40) included the potential risk of complicating processes and variation categories among countries being too different.

Table 1 provides a summary of the key recommendations from affiliates to improve Access. Recommendations were categorized into six areas (Table 1).

**Table 1:**
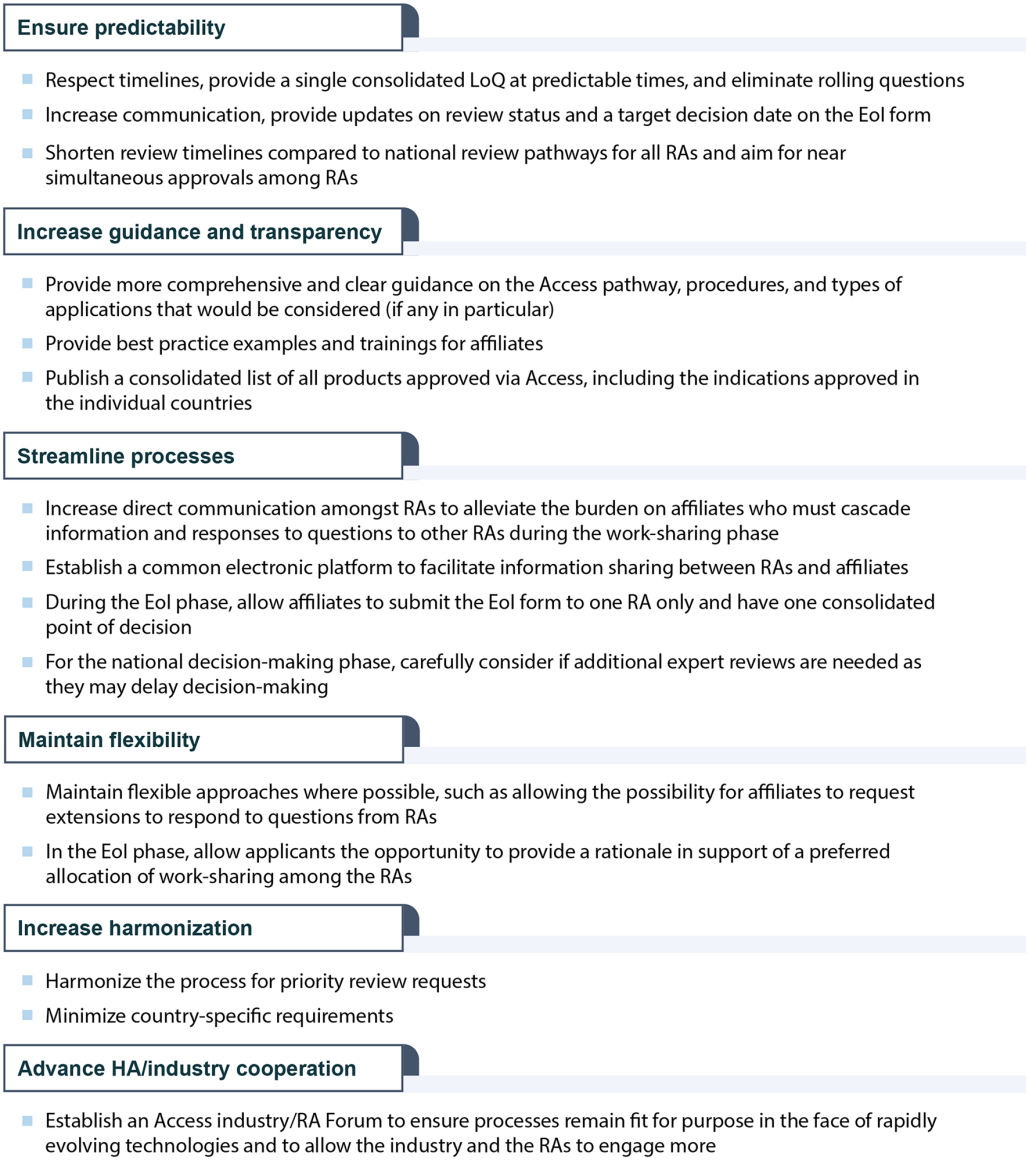
Summary of recommendations from affiliates to further improve Access

## Discussion

Overall, the survey results demonstrate that the perceptions and experiences of affiliates who had participated in Access were generally positive. Most of these affiliates were happy with their experience with the different phases of Access, their interactions with RAs, and appeared willing to continue to participate in the initiative.

Affiliates’ reasons for not having yet participated in Access included not having identified a suitable product candidate for the pathway (which could mean that some of these affiliates may be waiting for a good opportunity to participate in Access) and the perception that the process might be too complex to manage. Providing clear guidance and best practice examples may, therefore, encourage new members of the industry to participate in the initiative and further benefit some affiliates who have already taken part in Access. Lack of experience with the process was also given as a reason for not participating. Positively, results suggested that the more often affiliates participated in Access, the more experienced and comfortable they became with the process, as emphasized by the decreasing need to have a pre-submission logistics meeting after the first submission(s) for affiliates who have participated in the pathway. Raising awareness around this may further encourage participation from new applicants.

Most affiliates had good experiences with the different phases of Access, with many appreciating the good communication with RAs, and the receipt of a consolidated LoQ at set times. The Access pathway has improved over time, with improvements such as clearer guidance and more consolidated questions. However, challenges were also flagged, such as the receipt of rolling questions which negatively impacted resource planning for affiliates, delays in receiving feedback, and receipt of non-consolidated questions. Such differences in experiences might partly be due to participation in different Access reviews, with different submissions, contexts, and participating RAs, but it is also important to recognize that respondents may have taken part in Access at different times. Therefore, some responses may refer to experiences with early versions of the pathway when processes were less structured and clear. For example, in the EoI phase, some affiliates recommended RAs should provide updates on review status as well as a target decision date, improvements which some RAs have already made good progress on (from experience of some co-authors). Moreover, the joint pipeline meetings now offered by RAs to pharmaceutical and biotechnology companies can provide an opportunity for collaboration and information exchange on new developments, which may lead to further enhancements of the Access pathway.

Recommendations to improve Access were also suggested, as expected for a relatively recent initiative. Ensuring predictability is key for affiliates. Processes should be as predictable as possible to allow for appropriate management and use of affiliates’ limited resources. While the provision of consolidated LoQ at set times helped affiliates to properly plan and manage resources, rolling questions negatively impacted this ability and this might deter applicants from participating in the initiative. Rolling questions should therefore be eliminated. Discussions with RAs will be important to ensure alignment on this point as, while affiliates find rolling questions challenging, from the perspective of some RAs they may be perceived as accelerating the review process.

All affiliates, regardless of whether they had taken part in Access or not, would greatly value shorter review timelines with Access compared to national review pathways. This was not only considered the most important process improvement option by most affiliates who had taken part in Access, but it was also flagged as a required change to encourage participation in Access by the highest number of affiliates who had yet not participated in Access. Even though Access’ primary aim is not to shorten review timelines, but rather to facilitate efficient use of regulatory resources, shorter review timelines are probably still expected by the industry given individual RAs should have less work to do in an Access review compared to a standard national review. This improvement should certainly be balanced with allowing RAs enough time to conduct their assessments and providing affiliates with enough time to respond to queries from RAs.

Affiliates would benefit from more streamlined processes. Particularly, some found it challenging having to cascade information and responses to RAs and would welcome more direct communication between RAs. Gathering the perspective of RAs on this issue will be necessary to ascertain if there is a particular reason as to why RAs require the process to be conducted in this manner.

A single harmonized application for Access priority review was an important improvement according to all affiliates; better alignment of process for priority review applications was the second most selected required change by affiliates who had not yet participated in the pathway. Steps to enhance this aspect of Access were already taken after the survey was conducted, with the so called “Promise Pilot pathway” for priority review. In this new aligned process for priority review, including common timelines for the priority review request, the request is evaluated collaboratively with RAs seeking a consensus decision. The Promise Pilot pathway will be limited to NAS applications for products to diagnose, treat, or prevent serious, life-threatening, or severely debilitating conditions which have no current treatment available on the market [8]. In the future, Access might thus have enhanced positive impact, facilitating fast approvals of therapeutic products addressing areas of high unmet need.

Increased transparency would also be beneficial for affiliates. Despite some RAs having already published lists of products they have approved via Access, publication of one consolidated list of products approved via Access, accessible in the public domain and including the indications approved in the participating countries, would help affiliates to understand which types of therapeutic products have been approved to date and potentially aid in the planning of future submissions.

Establishing an industry/RA Forum would be a good opportunity to ensure continuous alignment between the pharmaceutical industry and RAs to ensure that processes can be revised or enhanced in a timely manner depending on the evolving environment and advancements in technology. Affiliates’ support of this initiative also indicates their willingness to increase engagement with the RAs and to help to drive improvements in Access. The feasibility of such a proposal will also need to be assessed in partnership with RAs, to understand their views on this.

Notably, the mention of gaining experience with an evolving work-sharing pathway, as a key benefit of Access for affiliates who had participated in the pathway, suggests that affiliates may be willing to continue to use this pathway, and that the number of new filings with this pathway might increase in the future. Furthermore, while the benefits of other regulatory frameworks such as Project Orbis are recognized, affiliates see great value in the work-sharing typical of Access, which allows for efficient use of RAs resources, encourages cooperation, and regulatory decision-making in a similar timeframe. Finally, 80% (*n* = 33) of affiliates who participated in Access thought that approval via “Access” had no impact on pricing and reimbursement timelines. This suggests that more might need to be done at the country levels to ensure that gains from earlier regulatory approvals translate into earlier access to innovative medicines for patients.

### Relevance for Future Policy Discussion

The insights and recommendations reported in this study should facilitate informed discussions on how to improve Access in the future, enhancing efficient use of resources and working practices for RAs and affiliates, reducing time for approval of innovative therapeutic products, and consequently contributing to faster access to medicines for patients. A future survey could explore the views of members of RAs on these topics. A dialogue between the industry and the RAs will be needed to realistically assess the feasibility of the recommendations offered in this study, which only considers the industry perspective. Insights and recommendations from this study, needs of RAs, and results of discussions among industry and RAs, should ideally inform the development of a potential future Access Consortium Strategic Plan (subsequent to the 2021–2024 Strategic Plan).

### Study Limitations

Insights included in this research article are solely gathered from the responses of affiliates of pharmaceutical companies that are members of the innovator trade associations (generics were outside the survey scope) in the five Access Consortium countries and may thus not necessarily be representative of the views of the whole industry. The experiences and perceptions of affiliates were mostly reported on as a whole, rather than by country to country, to capture the overall views on Access and to avoid making conclusions based on small sample sizes. When analyzing responses to open-ended questions (qualitative insights), some generalizations were made, and some concepts summarized. Only insights and recommendations mentioned often or deemed most relevant and/or interesting were reported, as opposed to all responses given by each survey respondent. The five Access Consortium RAs were not involved in the survey development nor in the review of the survey questions. The survey only captured industry perceptions (perceptions of RAs were not captured) around Access at the time of survey participation and should be considered as a starting point to inform policy discussions.

## Conclusion

The NASWSI is considered as a valuable pathway for the regulatory approval of NASs and indications by affiliates of the pharmaceutical companies that had participated in Access; with most affiliates happy about their experience, their interactions with RAs, and appearing willing to continue to participate in this initiative. Affiliates’ reasons for not having yet participated in Access included a lack of opportunity to do so and perceived barriers, such as the pathway being too complicated. Survey results indicated recent improvements in the Access pathway compared to earlier phases of the initiative, and the pipeline meetings offered by RAs to pharmaceutical and biotechnology companies may lead to further collaboration and enhancement of the Access pathway. Recommendations to improve Access can be summarized into six key areas: ensure predictability, increase guidance and transparency, streamline processes, maintain flexibility, increase harmonization, and advance RA-industry cooperation. These recommendations should facilitate informed discussions among relevant stakeholders as to how to improve Access to maximize efficiencies and accelerate approvals, therefore improving timely patient access to innovative medicines.
